# Azacytidine impairs NK cell activity in AML and MDS patients undergoing MRD-based pre-emptive treatment after allogeneic stem cell transplantation

**DOI:** 10.1038/bcj.2013.35

**Published:** 2013-08-30

**Authors:** C Schönefeldt, K Sockel, R Wehner, S Sopper, D Wolf, M Wermke, C Thiede, U Oelschlägel, G Ehninger, M Bornhäuser, U Platzbecker, M Schmitz

**Affiliations:** 1 Medical Clinic I, University Hospital Carl Gustav Carus, Dresden, Germany; 2 Institute of Immunology, Medical Faculty Carl Gustav Carus, Dresden, Germany; 3 University Hospital Innsbruck, Innsbruck, Austria; 4 Medical Clinic III, University Hospital Bonn, Bonn, Germany

We have recently demonstrated within a prospective trial (RELAZA1 study) that minimal residual disease (MRD)-triggered pre-emptive treatment with azacytidine (AZA) is able to prevent or at least delay hematologic relapse in patients with myelodysplastic syndromes (MDS) or acute myeloid leukemia (AML) after allogeneic hematopoietic stem cell transplantation (HSCT).^1^ Besides direct apoptotic effects at higher doses, AZA is considered to act as a hypomethylating agent at lower doses. Of note, hypomethylating effects not only affect cancer cells by inducing the re-expression of previously silenced tumor suppressor genes, but also influence gene expression in ‘bystander' cells, such as T lymphocytes (T cells). In line with this hypothesis, several groups recently reported epigenetic regulation of the FOXP3 locus, the critical transcription factor, driving regulatory T-cell differentiation. AZA-induced demethylation induced upregulation of FOXP3 and expansion of T-regulatory cells (Tregs), which might also contribute to lower graft-versus-host disease rates in the setting of allogeneic HSCT.^2^

Besides T cells, studies investigating the impact of AZA on other immune cell populations such as dendritic cells (DCs) or natural killer (NK) cells are rather limited. As AZA is currently explored in many clinical trials in the post-allograft setting, we aimed to study its impact on NK and DCs *in vitro* as well as *in vivo* when given as pre-emptive treatment within the RELAZA1 study.^1^ For *in vitro* experiments we used AZA doses of 100 nM, 1 μM and 3 μM, based on the observation of peak plasma levels of 1 μM in humans after the application of the standard 75 mg/m^2^ subcutaneous AZA dose.

First we investigated the impact of AZA on DCs, which are professional antigen-presenting cells, involved in the activation of innate and adaptive antitumor responses. Functional data revealed that 6-sulfo LacNAc (slan) DCs represent a major subset of native human blood DCs, which produce various proinflammatory cytokines and contribute to antitumor immunity by activating NK and T cells.^3, 4^ To explore how AZA modulates the secretion of proinflammatory cytokines by slanDCs *in vitro*, we isolated slanDCs immunomagnetically from peripheral blood of three healthy donors, at high purity (>90%) as described previously.^3^ Subsequently slanDCs were cultured with different concentrations of AZA (100 nM, 1 μM and 3 μM). After the addition of lipopolysaccharide (LPS, 1 μg/ml) for additional 18 h to stimulate cytokine secretion, supernatants were collected and secretion of proinflammatory cytokines was determined by enzyme-linked immunosorbent assay (ELISA). As demonstrated in Figure 1a–c, the production of tumor necrosis factor (TNF)-α, interleukin (IL)-6 and IL-12 by slanDCs was not altered by AZA. This is in accordance with data of a recent study, indicating that AZA does not influence the release of the above-mentioned proinflammatory cytokines.^5^ But in contrast, AZA significantly impaired the secretion of IL-27, which is known to be inversely correlated with Th17 cell activation.

In further experiments, we evaluated whether AZA alters the capacity of slanDCs to stimulate T-cell responses. For this purpose, slanDCs were again exposed to different concentrations of Aza (100 nM, 1 μM, 3 μM) and co-cultured with immunomagnetically isolated allogeneic CD4^+^ T cells for 7 days. Proliferation was determined by ^3^H-thymidine incorporation. Again, the obtained results revealed no significant effect of AZA on slan DC-induced T-cell proliferation (Figure 1d). This is in agreement with a previous finding, indicating that AZA at a concentration of 4 μM does not alter the capacity of monocyte-derived DCs to stimulate T-cell proliferation.^5^

Next we explored the impact of AZA on NK cells, which have an important role in the regulation of immune responses against malignant cells. To investigate the impact of AZA on the immunomodulatory ability and cytotoxic potential of NK cells, human CD56^+^ CD3^−^ NK cells were isolated immunomagnetically from freshly prepared peripheral blood mononuclear cells of healthy donors at a purity of >90% as assessed by flow cytometry. Isolated NK cells of each donor were then exposed *in vitro* to increasing concentrations of AZA (100 nM, 1 μM, 3 μM) in the presence of the activating cytokine IL-2 (300 U/ml) for 5 days. Interestingly, AZA significantly impaired IFN-γ release by IL-2-activated NK cells (Figure 2a). To explore the cytotoxic potential of NK cells we used the chromium release assay^6^ with the tumor cell line K562 as target cells. As depicted in Figure 2b, AZA efficiently inhibited the cytotoxic activity of NK cells at a concentration of 3 μM. These results are in agreement with a previous report, indicating that AZA suppresses the cytolytic potential of a human NK cell line as well as native human NK cells.^7^ To get insights into the underlying mechanisms, we explored whether AZA modulates the viability of NK cells. As demonstrated in Figure 2c, AZA increased the percentage of apoptotic NK cells at a concentration of 3 μM. Further more, we investigated the impact of AZA on the expression of important NK cell receptor and cytotoxic molecules, given the fact, that some NK cell receptors are regulated by promoter methylation of the respective genes.^7, 8^ Notably, AZA reduced the surface density of TRAIL and the activating receptors NKG2D and NKp46 on NK cells (Figure 2d–f). In contrast, the expression of NKp30, NKp44, perforin, granzyme-B or Fas ligand was not altered by AZA (data not shown).

To further substantiate these *in vitro* findings, we investigated the effect of AZA on the expression of NK cell-activating receptors and cytotoxic molecules *in vivo* within the prospective RELAZA trial (NCT00422890).^1^ This clinical study investigated whether pre-emptive AZA treatment (75 mg/m^2^ for 7 days) in MDS or AML patients with MRD is a suitable tool to prevent hematologic relapse. In agreement with our *in vitro* results, we found a significantly reduced expression of TRAIL, NKG2D or NKp46 on NK cells during AZA therapy in comparison to NK cells obtained before treatment (Figure 2g–i) considering eight patients. Respectively, the observed reduction of NK cell-activating receptors and TRAIL during AZA treatment was seen in patients with a reduction or stable course of MRD, suggesting that the clinical effect of AZA is not mediated by NK cell activation.

In summary, despite the limited sample sizes we demonstrated that AZA does not modulate the potential of human slanDCs to produce proinflammatory cytokines and promote proliferation of CD4^+^ T cells. These results indicate that immunostimulatory properties of slanDCs, which may have an important role in tumor cell elimination are maintained by AZA. Further studies revealed that AZA efficiently inhibits IFN-γ secretion and reduces cytotoxic potential of activated human NK cells. This impaired NK cell function might be explained by the potential of AZA to increase NK cell apoptosis and reduce the surface expression of the cytotoxic effector molecule TRAIL as well as the activating receptors NKG2D and NKp46 on NK cells *in vitro* and *in vivo*. Taken together these data suggest that the clinical effects of AZA in the post-transplant setting are not mediated by enhancing NK cell activity. Instead, the data indicate an inhibitory effect of AZA on NK cell function. Until the mechanisms of AZA are not fully understood, patients should be carefully selected to receive hypomethylating agents in the post-transplant setting and therapy should be given within controlled clinical trials.

## Figures and Tables

**Figure 1:**
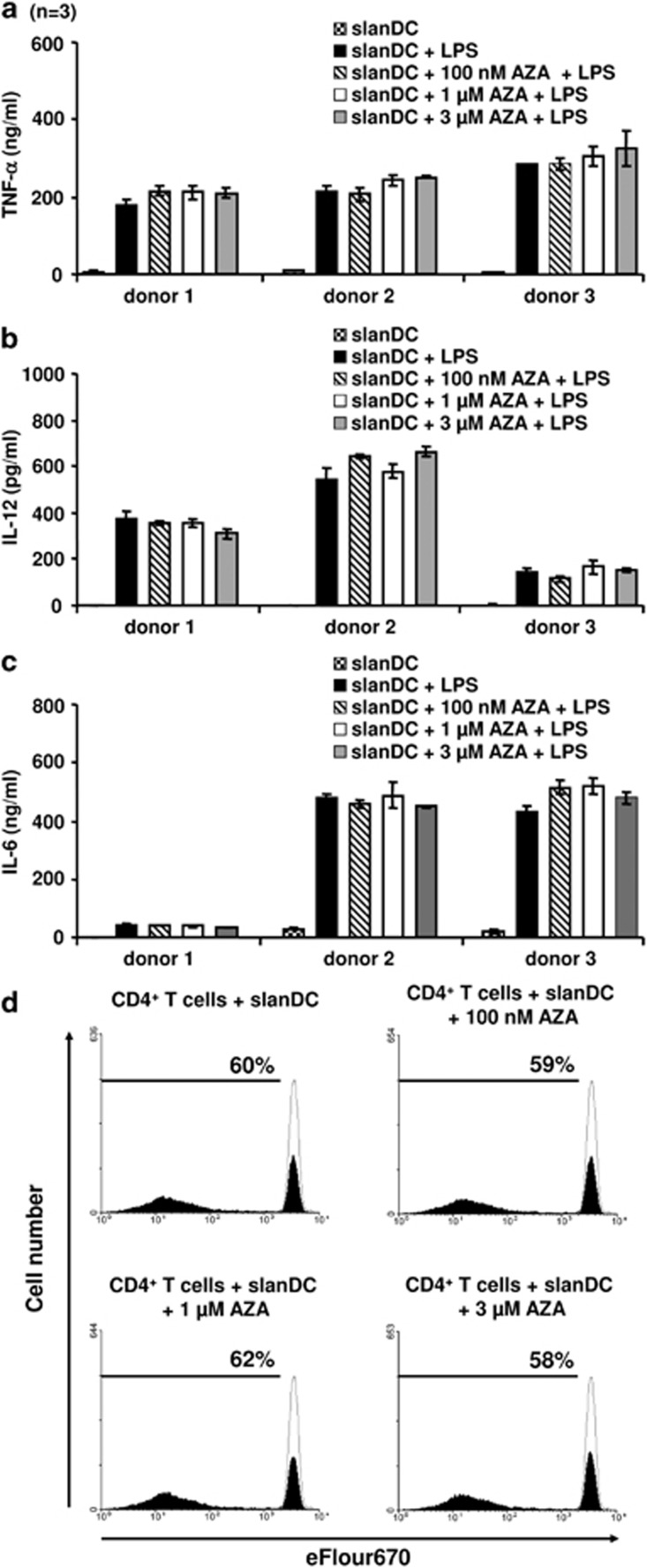
Impact of AZA on immunostimulatory properties of slanDCs. (**a**–**c**) Freshly isolated slanDCs from three healthy donors were cultured in the presence or absence of 100 nM, 1 μM or 3 μM AZA. SlanDCs were maintained for 6 h and then activated by adding LPS. After additional 18 h, supernatants were collected and the concentration of TNF-α (**a**), IL-12 (**b**) and IL-6 (**c**) was determined by ELISA. Columns represent means±s.e. of results obtained from three different healthy donors. For each donor the mean of triplicate determination was used. (**d**) Freshly isolated slanDCs were cultured for 6 h with or without 100 nM, 1 μM or 3 μM AZA, washed, and coincubated with eFluor670-stained allogeneic CD4^+^ T cells. After 7 days, proliferation of CD4^+^ T cells was analyzed by flow cytometry. Values represent the percentage of proliferating cells (filled) compared to non-activated cells (empty).

**Figure 2:**
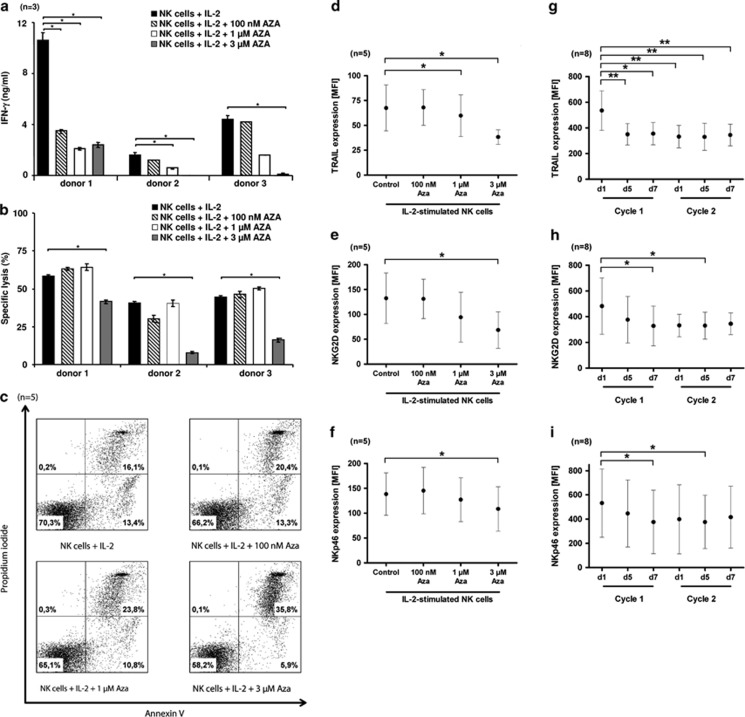
Influence of AZA on NK cell activation. (**a**–**f**) Freshly isolated NK cells from up to five healthy donors were incubated with or without 100 nM, 1 μM or 3 μM AZA and IL-2 for 5 days. (**a**) Supernatants were harvested and INF-γ production of NK cells was determined by ELISA. The results are presented as means±s.e. of duplicate determinations. Asterisks indicate a statistically significant difference (*P*≤0.05), *n*=3. (**b**) AZA-treated and non-treated IL-2-stimulated NK cells were co-cultured with ^51^Cr-labeled K-562 cells at an E/T ratio of 1:1. After 4 h of incubation, chromium release was measured. The results are presented as means±s.e. of triplicate determinations. Asterisks indicate a statistically significant difference (*P*≤0.05), *n*=3. (**c**) AZA-treated and non-treated IL-2-stimulated NK cells were stained using annexin V and propidium iodide to determine apoptotic cell death by flow cytometry. The percentages of cells positive for annexin V and/or propidium iodide are indicated in the dot blots as well as the percentages of cells being negative for both agents. The results of one representative donor out of five performed with similar results are demonstrated. (**d**–**f**) The expression levels of TRAIL, NKG2D and NKp46 of AZA-treated and non-treated IL-2-stimulated NK cells were determined by flow cytometry. Each dot represents the mean of mean fluorescence intensity (MFI) for each surface molecule obtained from NK cells of five healthy donors. Error bars represent the standard deviation. Asterisks indicate a statistically significant difference (*P*≤0.05). (**g**–**i**) NK cells were obtained from AML or MDS patients after HSCT before (d1=day 1), during (d5) and at the end (d7) of AZA treatment cycles 1 and 2. Subsequently, expression levels of TRAIL, NKG2D and NKp46 were measured by flow cytometry. Each dot represents the mean of MFI for each surface molecule obtained from NK cells of eight patients. Error bars represent the standard deviation. Asterisks indicate a statistically significant difference (*P*≤0.05).

## Notes

UP has received speakers honoraria and research funding from Celgene. The remaining authors declare no conflict of interest.

## References

[bib1] Platzbecker U Wermke M Radke J Oelschlaegel U Seltmann F Kiani A Azacitidine for treatment of imminent relapse in MDS or AML patients after allogeneic HSCT: results of the RELAZA trial Leukemia 2012 26 381 389 21886171 10.1038/leu.2011.234 PMC3306138

[bib2] Goodyear OC Dennis M Jilani NY Loke J Siddique S Ryan G Azacitidine augments expansion of regulatory T cells after allogeneic stem cell transplantation in patients with acute myeloid leukemia (AML) Blood 2012 119 3361 3369 22234690 10.1182/blood-2011-09-377044

[bib3] Schakel K von Kietzell M Hansel A Ebling A Schulze L Haase M Human 6-sulfo LacNAc-expressing dendritic cells are principal producers of early interleukin-12 and are controlled by erythrocytes Immunity 2006 24 767 777 16782032 10.1016/j.immuni.2006.03.020

[bib4] Schmitz M Zhao S Deuse Y Schakel K Wehner R Wohner H Tumoricidal potential of native blood dendritic cells: direct tumor cell killing and activation of NK cell-mediated cytotoxicity J Immunol 2005 174 4127 4134 15778372 10.4049/jimmunol.174.7.4127

[bib5] Frikeche J Clavert A Delaunay J Brissot E Gregoire M Gaugler B Impact of the hypomethylating agent 5-azacytidine on dendritic cells function Exp Hematol 2011 39 1056 1063 21856273 10.1016/j.exphem.2011.08.004

[bib6] Baltz KM Krusch M Bringmann A Brossart P Mayer F Kloss M Cancer immunoediting by GITR (glucocorticoid-induced TNF-related protein) ligand in humans: NK cell/tumor cell interactions FASEB J 2007 21 2442 2454 17360848 10.1096/fj.06-7724com

[bib7] Gao XN Lin J Wang LL Yu L Demethylating treatment suppresses natural killer cell cytolytic activity Mol Immunol 2009 46 2064 2070 19394699 10.1016/j.molimm.2009.02.033

[bib8] Chan HW Kurago ZB Stewart CA Wilson MJ Martin MP Mace BE DNA methylation maintains allele-specific KIR gene expression in human natural killer cells J Exp Med 2003 197 245 255 12538663 10.1084/jem.20021127 PMC2193817

